# Medial temporal lobe function during emotional memory in early Alzheimer’s disease, mild cognitive impairment and healthy ageing: an fMRI study

**DOI:** 10.1186/1471-244X-13-76

**Published:** 2013-03-06

**Authors:** Mario A Parra, Vivek Pattan, Dichelle Wong, Anna Beaglehole, Jane Lonie, Hong I Wan, Garry Honey, Jeremy Hall, Heather C Whalley, Stephen M Lawrie

**Affiliations:** 1 Scottish Dementia Clinical Research Network, Human Cognitive Neuroscience and Centre for Cognitive Ageing and Cognitive Epidemiology, University of Edinburgh, Edinburgh EH8 9JZ, UK; 2 Higher Specialty Trainee -Old Age Psychiatry, Stobhill Hospital, Glasgow, UK; 3 Division of Psychiatry, School of Molecular Medicine, Royal Edinburgh Hospital, Morningside, University of Edinburgh, Edinburgh, EH10 5HF, UK; 4 Old Age Psychiatry, St John’s Hospital, West Lothian, UK; 5 Translational Medicine, BioTherapeutics Clinical Programs, Pfizer, Inc, Cambridge, USA

**Keywords:** Alzheimer’s disease, Mild cognitive impairment, fMRI, Medial temporal lobe, Incidental memory, Emotional memory

## Abstract

**Background:**

Relative to intentional memory encoding, which quickly declines in Mild Cognitive Impairment (MCI) and Alzheimer’s disease (AD), incidental memory for emotional stimuli appears to deteriorate more slowly. We hypothesised that tests of incidental emotional memory may inform on different aspects of cognitive decline in MCI and AD.

**Methods:**

Patients with MCI, AD and Healthy Controls (HC) were asked to attend to emotional pictures (i.e., positive and neutral) sequentially presented during an fMRI session. Attention was monitored behaviourally. A surprise post-scan recognition test was then administered.

**Results:**

The groups remained attentive within the scanner. The post-scan recognition pattern was in the form of (HC = MCI) > AD, with only the former group showing a clear benefit from emotional pictures. fMRI analysis of incidental encoding demonstrated clusters of activation in para-hippocampal regions and in the hippocampus in HC and MCI patients but not in AD patients. The pattern of activation observed in MCI patients tended to be greater than that found in HC.

**Conclusions:**

The results suggest that incidental emotional memory might offer a suitable platform to investigate, using behavioural and fMRI measures, subtle changes in the process of developing AD. These changes seem to differ from those found using standard episodic memory tests. The underpinnings of such differences and the potential clinical use of this methodology are discussed in depth.

## Background

Memory and emotion are closely related to the functions of the Medial Temporal Lobe (MTL) and both are affected by Alzheimer’s disease (AD). Episodic memory declines remarkably in patients with AD and in those with Mild Cognitive Impairment (MCI) [1-5]. The impact of AD on structures such as the hippocampus and the amygdala appears to be a responsible mechanism [6-11]. Functional Magnetic Resonance Imaging (fMRI) studies have suggested that AD-related changes seem to start very early and might express initially as a functional reorganization of MTL regions during episodic memory tasks [6].

There seems to be a relationship between the episodic memory impairment observed in patients with MCI and AD and their difficulties in processing emotional information. Emotional processing has been found to be sensitive to the effects of AD [12-17]. Patients with amnestic MCI and AD present with difficulties to process emotional information in experimental and real life contexts [13,18-21]. Emotion is thought to support the formation of stable and durable representations of past experiences in long term memory [22]. Dere et al. [22] suggested that a deafferentiation of the hippocampus from emotional input, which might be required for episodic memory formation, could explain the development of memory and learning impairments throughout the course of AD [15]. The episodic memory impairment observed in MCI and early AD using explicit memory tasks frequently overlaps [23,24] albeit not all MCI patients will progress to AD. Therefore, the actual mechanisms underlying memory decline in these two conditions need further investigation. This knowledge can inform theoretical models which could help develop new clinical methods.

Functional measures of brain activity during incidental memory processing of emotional stimuli may offer a useful approach to address this issue. Incidental or implicit memory is commonly assessed by presenting participants with list of items (e.g., pictures, words, etc.) which they do not need to remember, and ask them later in a surprise test to recall or recognise the previously presented materials. The combination of emotional processing during implicit memory tests has already proved a valid approach to investigate whether episodic memory encoding can be enhanced in population with psychiatric disorders [25] (see also [26] for a recent meta-analysis on this methodology). Implicit memory seems to deteriorate much slower in the course of AD than explicit memory. This may offer opportunities to identify clear cut measures of AD progression. For example, MCI patients have shown preserved implicit learning functions in the presence of deteriorated explicit recognition [23]. Golby et al. [27] used fMRI to investigate explicit and implicit memory for natural scenes in AD. AD patients showed impaired explicit recognition memory but intact implicit memory for the scenes. The dissociation between impaired explicit and preserved implicit memory was found to be accounted for by encoding impairment in MTL and fusiform regions with intact implicit encoding in earlier-stage occipital cortex (see also [28-33] for evidence in support to this proposal), thus suggesting that AD impacts on the processing of emotion-related information differently depending on whether explicit/intentional or implicit/incidental mechanisms are used to perform the task.

Based on the aforementioned literature, one might predict that functional assessment of MTL structures (e.g., parahippocampal gyrus - PHG, hippocampus, and amygdala) during incidental emotional memory may shed light into the differential impact of MCI and AD on these brain regions [34-36]. We are not aware of previous studies that combined these approaches to address this issue. The present study investigated this hypothesis in a sample of AD and MCI patients and in Healthy Controls. To this aim we chose a task previously reported by Whalley et al. [25] which investigates emotional memory for positive and neutral scenes using an incidental memory paradigm. We predicted a more evident decline in functional and behavioural measures of incidental memory for emotional stimuli in AD relatively to MCI patients and Healthy Controls. Based on previous structural and functional neuroimaging studies (e.g., [15,17,25]), we predicted differential activation of MTL structures between the groups.

## Methods

### Participants

In total 35 individuals were recruited. Three participants (one from each group) requested that the scan be stopped before completion of the emotional memory paradigm and hence provided no functional imaging data. Of the 32 individuals remaining, two subjects were subsequently excluded from analysis, one because of excessive movement during the scan (>3 mm per TR) and one due to excessive ventricular size. The research groups comprised 10 patients with MCI, 10 patients with AD and 10 age-matched Healthy Controls (HC). The AD subjects were initially classified in accordance with the National Institute of Neurologic, Communicative Disorders and Stroke - AD and Related Disorders Association (NINCDS-ADRDA, [37]). Patients were recruited from tertiary referrals to the neuropsychological service or via referrals to the old age psychiatry service at the Royal Edinburgh Hospital. The MCI participants were recruited from tertiary referrals to the local neuropsychological assessment service for older adults and met criteria for amnestic MCI [38]. HC were recruited from the cohort study by Lonie et al. [39]. Six HC, 4 MCI patients and 3 AD patients were taking antihypertensive medications, 2 MCI patients and 6 AD patients were taking Acetylcholinesterase inhibitors.

Exclusion criteria for AD patients were other medical/neurological conditions which could account for memory loss, untreated depressive illness, significant cerebrovascular disease on neuroimaging (i.e. exclusion of probable vascular aetiology) and significant motor or visual problems, or other factors which would preclude MRI scanning. Standard operational criteria for amnestic MCI were (1) memory complaint, corroborated by an informant; (2) abnormal memory function defined by memory performance of 1 standard deviation or more below age means on the delayed recall of one paragraph from the Logical Memory II subtest of the Wechsler Memory Scale–Revised [40]; (3) normal general cognitive function, as determined by a clinician’s judgment based on a structured interview with the patient and an informant and a Mini-Mental State Examination (MMSE) score greater than or equal to 24; (4) no or minimal impairment in activities of daily living (ADLs), as determined by a clinical interview with the patient and informant; and (5) not sufficiently impaired, cognitively and functionally, to meet DSM-IV criteria for AD, as judged by an experienced AD clinician. We decided to use a less stringent threshold to assess objective cognitive decline in MCI (1 rather than 1.5 SD, but see [41] for recent guidelines) as we were interested in very early features of preclinical AD. Based on recent evidence [42], such boundaries may be difficult to delineate if higher cut-off scores are applied. Evidence from recent longitudinal studies supports the validity of this methodological approach [39]. The exclusion criteria were as described above. For the purposes of the current study to determine if any of the MCI patients should be classified as AD, or had returned to normal, a case summary was prepared describing clinical notes, current ACE and MMSE scores, and further background information as required, to determine current diagnostic status. Finally, the exclusion criteria for HC were a history of medical, psychiatric or neurological conditions that could affect cognitive functioning. HC group was matched to the MCI and AD groups in terms of age and gender. All the participants recruited into the study gave their informed consent. The study protocol (ref. 06/S1102/51) was reviewed and approved by the Lothian Local Research Ethics Committee 2, NHS. The demographic and psychometric characteristics of these groups are presented in Table 1.

**Table 1:** Demographic and psychometric characteristics of the three groups of participants

	**Controls (n = 10)**	**MCI (n = 10)**	**AD (n = 10)**	**ANOVA**		**Bonferroni-corrected post-hoc tests ( *p-values *)**		
	**M(SD)**	**M(SD)**	**M(SD)**	***F***	***p-values***	**HC vs. MCI**	**HC vs. AD**	**MCI vs. AD**
**Age (years)**	74 (8.89)	76 (9.03)	78 (7.56)	0.57	*ns*	*ns*	*ns*	*ns*
**Gender (M:F)**	4:6	3:7	5:5	*(Chi-square = ns)*		*ns*	*ns*	*ns*
**ACE-R score**	94.80 (4.54)	86.50 (5.50)	72.10 (10.35)	27.42	< 0.001	0.043	0.000	0.000
**MMSE**	29.10 (1.60)	27.50 (2.22)	23.60 (3.37)	15.35	< 0.001	0.357	0.000	0.002
Attention and Orientation (*)	17.60 (0.97)	17.10 (1.10)	14.80 (2.20)	9.57	0.001	1.000	0.001	0.007
Memory (*)	23.90 (2.23)	17.90 (3.96)	11.30 (4.37)	29.96	0.000	0.003	0.000	0.001
Executive Functions (*)	12.70 (1.49)	11.00 (1.33)	8.20 (2.66)	13.98	0.000	0.175	0.000	0.009
Language (*)	25.60 (0.70)	24.90 (1.60)	23.10 (2.51)	5.33	0.011	1.000	0.011	0.092
Visuospatial functions (*)	15.40 (0.70)	15.60 (0.52)	14.10 (1.66)	5.65	0.009	1.000	0.037	0.014
**NART**	118 (7.70)	116 (6.88)	108 (10.60)	4.35	0.028	1.000	0.024	0.148

(*) Sub-scores of ACE-R. The minimum educational qualification achieved by the participants recruited into the study was high-school.

### Scanning procedure

Imaging was carried out at the Brain Imaging Research Centre (BIRC) for Scotland on a GE 1.5 T Signa scanner (GE Medical, Milwaukee, USA). The imaging protocol consisted of a localizer scan, followed by a T2-weighted fast spin-echo sequence, a structural T1 weighted sequence and a functional imaging paradigm: an emotional memory scan. The emotional memory task involved axial gradient-echo planar images (EPI) (TR/TE = 2500/40 ms; matrix = 64 × 64; field of view (fov) = 24 cm) acquired continually over two runs. Thirty contiguous interleaved 5-mm slices were aligned to the anterior and posterior commissure within each TR period. Each acquisition consisted of 99 volumes, of which the first four volumes were discarded. The T1 sequence yielded 128 contiguous 1.2 mm coronal slices (matrix = 192 × 192; fov = 24 cm; flip angle 8°).

### Emotional memory paradigm

The paradigm was that previously reported by Whalley et al. [25]. To construct the paradigm 72 images were selected from the International Affective Picture System [43], comprising 36 images depicting emotionally neutral scenes and 36 depicting emotionally positive scenes. Images were selected based on their valence and arousal. Mean normative control ratings for emotional valence (scored from 1 to 9 for most positive) and emotional arousal (scored from 1 minimum to 9 maximum) for the positive scenes were: 7.30 (std dev 0.52) and 5.72 (std dev 0.62) for set 1, and 7.12 (std dev 0.49) and 5.67 (std dev 0.62) for set 2. Images were divided in two sets for counterbalancing purposes (i.e., some participants were presented with set 1 within the scanner and set 2 was used as a distracter in the post-scan test and others received the opposite order). Emotional valence and emotional arousal ratings for the neutral scenes were 5.05 (std dev 0.47) and 3.07 (std dev 0.49) for set 1 and 5.14 (std dev 0.50) and 3.01 (std dev 0.59) for set 2. Similar valence and arousal values have been found to elicit significantly different responses across emotional categories [44,45].

The scanning session was divided into blocks during which images of positive emotional scenes (emotion blocks), images of neutral emotional scenes (neutral blocks), and baseline fixation periods (baseline blocks) were presented. The baseline condition comprised the presentation of a fixation cross in the centre of the screen. Subjects completed two runs of the task each comprising 3 emotion blocks, 3 neutral blocks (25 s duration each) and 7 interleaved blocks of the baseline (12.5 s duration). For the emotion and neutral blocks six colour images were presented for 4 s each in random order and subjects were asked to simply press a button when they had seen the picture. This procedure was aimed at keeping arousal high within the scanner and to ensure that the participants were encoding the information presented. Was precisely this memory process (i.e., encoding) the one we investigated with this incidental memory paradigm^a^. Subjects were given written and verbal instructions prior to scanning. In these instructions they were not asked to remember the presented images but just to press a button whenever a new image appeared on the screen. For the scanning component the delivery of stimuli was conducted using Presentation software.

Immediately after the scan a post-scan surprise recognition test was conducted on a laptop using E-Prime software. For the post-scan recognition memory test a further matched 72 images were selected as distracters following the counterbalancing procedure described above. Participants were then shown 144 slides (72 old items and 72 new distracters) and were asked to indicate whether the images have been seen within the scanner (‘recognised’) or whether they were new images (‘not recognised’). They were asked respond by pressing two buttons previously assigned to each response. To further counterbalance old and new images and the order of presentation of the conditions, four parallel versions of the task were used, sets 1 and 2 with either neutral (e.g., set 1.1 or 2.1) or positive scenes (e.g., set 1.2 or 2.2) presented first. Pen and paper tests including the National Adult Reading Test (NART) of verbal IQ [46] and Addenbrooke’s Cognitive Examination (ACE) [47] were also conducted after the scanning session.

### Behavioural analysis

Within-scanner we recorded the number of responses to the stimuli. For the post-scan test, recognition memory was computed based on the percentage of corrected recognition (Hits – False alarms). Moreover, we implemented the Signal Detection Theory to calculate whether poor sensitivity (A’) or a response bias (β) could account for group differences in performance. Poor sensitivity would be informative of difficulties keeping signal (i.e., positive images) and noise (i.e., neutral images in memory) separate in memory. A negative response bias would indicate a tendency to choose the option “not seen before” in the post-scan recognition task, whereas a positive bias would reflect a tendency to choose the option “seen before”. Statistical tests were conducted using SPSS for Windows (version 18.0, SPSS Inc., USA). Analysis of Variance (ANOVA) was used to test for differences between groups. When the dependent variable was not normally distributed, Kruskal-Wallis non-parametric statistics was used. Bivariate Pearson Correlation analysis with behavioural and neuroimaging measures was also performed. This was aimed at identifying possible patterns of correlation between performance in the post-scan memory test and brain activation during incidental encoding in the region that proved most sensitive in group comparisons (i.e., MTL).

### Image processing for emotional memory paradigm

EPI and T1 images were reconstructed offline into NIFTI format (Mayo Foundation, Rochester, MN, USA) using DICOM convert functions available in SPM5 (Statistical Parametric Mapping: The Wellcome Department of Cognitive Neurology and collaborators, Institute of Neurology, London) running in Matlab (The MathWorks, Natick, MA, USA). To assess data quality reconstructed images were examined using ‘Art Repair’ software (Centre for Interdisciplinary Brain Sciences Research, Stanford University, USA, http://cibsr.stanford.edu/tools/human-brain-project/artrepair-software.html). For preprocessing, images were corrected for differences in image acquisition time between slices (slice timing) and then realigned to the mean functional image using a two-pass procedure to correct for movement artefact throughout the period of image acquisition. Normalization was performed to the EPI template available within SPM5 using standard preprocessing routines.

### fMRI within-groups analyses

This was conducted using standard procedures using the general linear model approach as implemented in SPM5. At the individual participant level the data was modelled with the two conditions (emotion and neutral) each modelled by a boxcar function convolved with a synthetic haemodynamic response function. Estimates of head movement from the realignment stage of pre-processing were included as additional regressors in the model. Before fitting the model, the participants data was filtered in the time domain using high pass filter (128 s cut-off) and serial correlations were accounted for by using the autoregressive (AR(1)) model. All pre-processing and analysis was conducted using default settings unless otherwise stated. Contrast images for each participant were then constructed for (i) all scenes versus baseline, (ii) emotional scenes versus baseline, (iii) neutral scenes versus baseline and (iv) emotional scenes versus neutral scenes.

### fMRI between-groups analyses

Contrast images were then entered into a second level random effects analysis to examine areas of activation within each of the three groups (one sample t test), and differences in activation between the groups (ANOVA). Between group statistical maps were thresholded at a level of p = 0.001 uncorrected, and regions were considered significant at the cluster corrected threshold of p < 0.05 (K*e* = 10 voxels). The latter is corrected for the chance of finding a cluster of this extent within the search volume and is therefore not reported in terms of FWE or FD. Based on our region of primary interest a small volume correction was used for MTL regions (amygdala, hippocampus and parahippocampal gyrus) based on the WFU PickAtlas [48,49].

## Results

### Demographics and psychometric variables

There were no significant differences between the groups in terms of age or gender, although there was a significant difference in NART IQ, and as expected, a significant difference in ACE and MMSE scores whereby AD patients scored more poorly than MCI and HC on these tests (Table 1). When the different subscores of ACE entered ANOVA, MCI patients showed a primarily amnestic deficit which persisted after controlling for IQ. AD patients however, showed significant deficits in all the subscores of ACE-R relative to both HC and MCI. For all the between group analyses presented below, IQ and gender were entered as covariates.

### Behavioural measures

**Figure 1:**
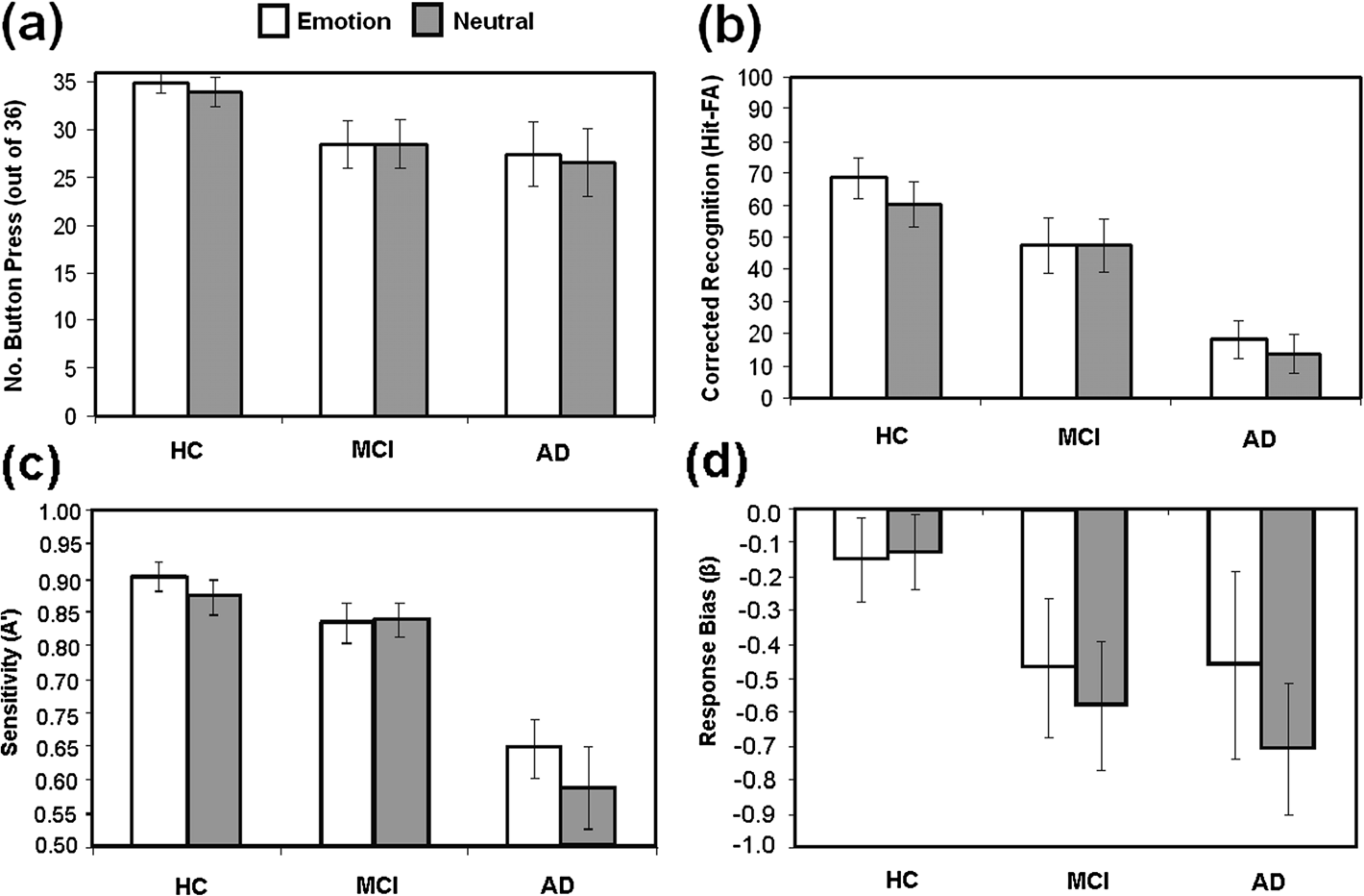
**Behavioural data from the scanner and post-scanner emotional memory tests from the three groups of participants (error bars represent the standard errors of the mean).** (**a**) Number of button presses while participants observed the images within the fMRI scanner. (**b**) Corrected recognition in the post-scan memory test. (**c** &**d**) Variables of the Signal Detection Theory which inform on sensitivity (**c**) and response bias (**d**).

A two-way mixed ANOVA with Group as the between-subjects factor (HC vs. MCI vs. AD) and Block (Emotion vs. Neutral) as the within-subjects factor was first performed with the number of button press within the scanner. Mean data are shown in Figure 1a. There was no effect of Group [F(2,27) = 2.29, n.s.] or Block [F(1,27) = 1.93, n.s.] nor was there a significant interaction between these factors [F(2,27) = 1.71, n.s.]. A second ANOVA was carried out using the same model to analyse performance on the post-scan memory test. Mean data are shown in Figure 1b. There was a significant effect of Group [F(2,27) = 12.05, p < 0.001] whereby AD patients performed more poorly than both MCI (Mean Difference – MD = 31.66, p < 0.05) and HC (MD = 48.61, p < 0.001). These last two groups did not significantly differ (MD = 16.94, p = 0.292). The effect of Block was also found to be significant [F(1,27) = 8.96, p = 0.006] whereby the Emotion block resulted in better post-scan performance than the neutral block (MD = 4.26, p = 0.009). The Group by Block interaction tended towards significance [F(2,27) = 2.87, p = 0.07].

Further post-hoc tests were performed to explore this tendency in more depth. Paired-sample t-tests carried out across blocks for each group separately confirmed that HC were the only group that showed an effect of emotion on performance whereby Emotional scenes resulted in better incidental memory than neutral scenes (HC: MD = 8.33, t = 3.87, p = 0.004; MCI: MD = 0.002, t = 0.001, n.s.; AD: MD = 4.44, t = 1.58, n.s.).

The analysis using the Signal Detection Theory showed a significant effect of Group when A’ was compared across Neutral images [Kruskal-Wallis test = 13.41 df = 2, p = 0.001] and Positive images [Kruskal-Wallis test = 15.2 df = 2, p = 0.001] (Figure 1c). AD patients were less able to keep separate signal from noise in memory relative to HC [Positive = 11.15, p < 0.001; Negative = 13.5, p = 0.005] and to MCI patients [Positive = 9.6, p = 0.044; Neutral = 11.1, p = 0.001]. More negative response bias (β) was observed for AD and MCI patients than for HC (Figure 1d). Neutral images tended to increase this negative bias in these groups. However, no significant effects were observed during the analysis of this variable.

### fMRI measures: within-group analyses

**Figure 2:**
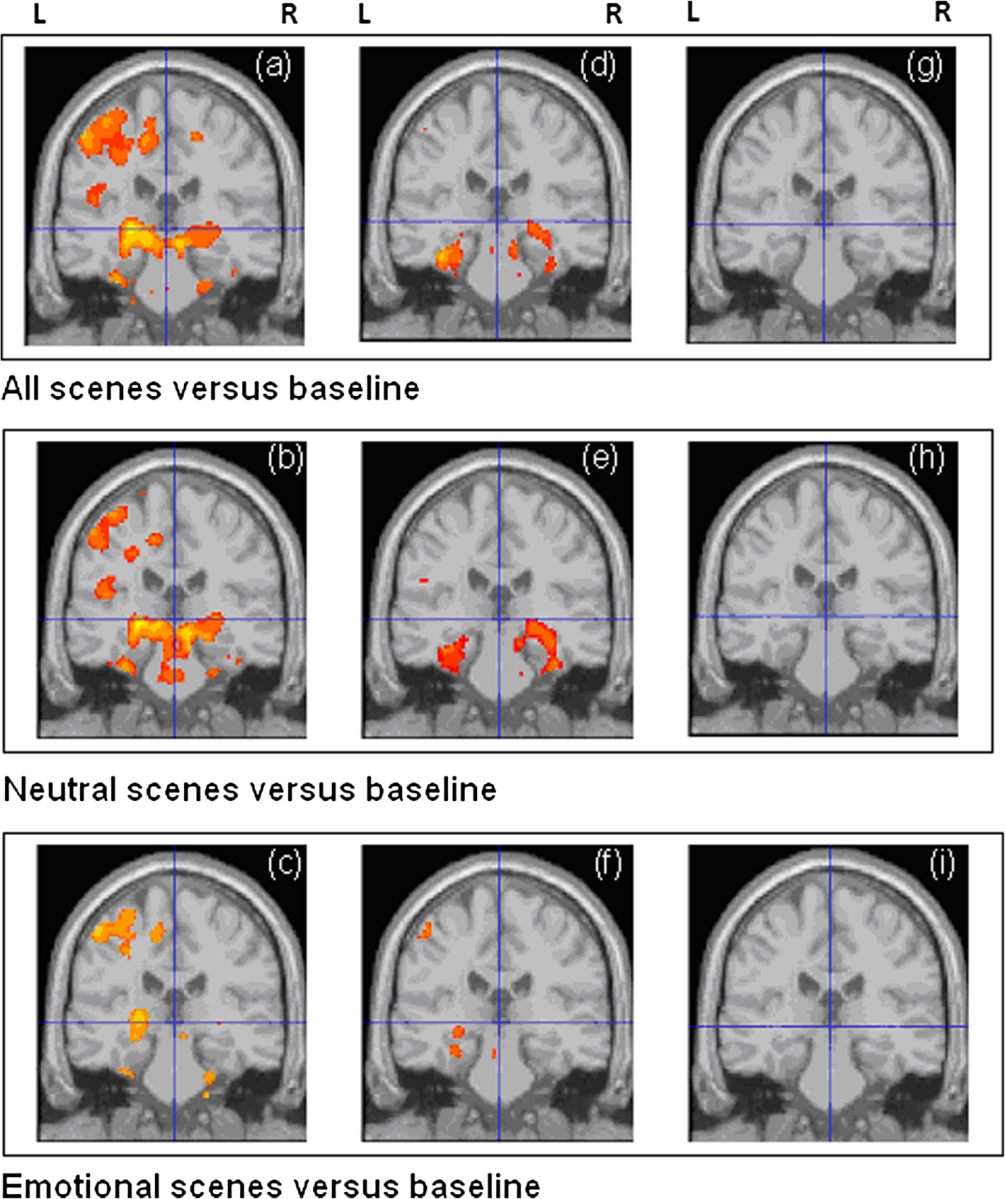
**Within group activation maps for HC (a, b, c), MCI patients (d, e, f) and AD patients (g, h, i).** Images thresholded at p = 0.01 uncorrected for illustration purposes only, all statistical comparisons performed at p = 0.001 uncorrected threshold, as described in methods section.

Within group activations are presented in Figure 2 (for a fuller description see Additional file 1: Figure S1 and S2). For the within-group analyses with all scenes versus baseline, the HC and MCI group both demonstrated clusters of activation in MTL regions which were not observed in the AD group. Other regions of activation in HC and MCI group included medial frontal regions, insula, fusiform, cerebellum and occipital cortex. There was less activation overall in the AD group, but clusters were observed in fusiform, cerebellum and occipital regions. A similar pattern of findings was seen for the contrasts of emotional scenes versus baseline and neutral scenes versus baseline. However, the contrast of emotional scenes versus neutral scenes in controls did not yield significant differences.

### fMRI measures: between-group analyses

**Figure 3:**
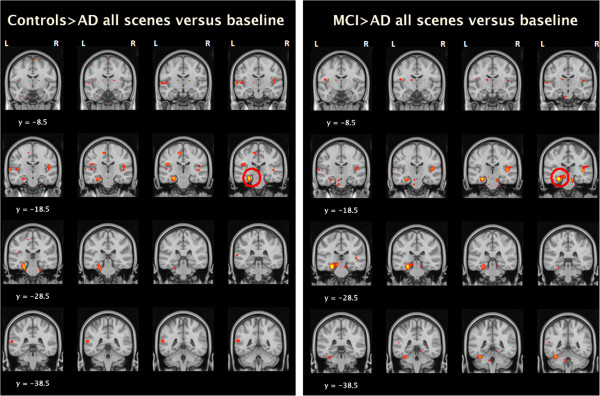
Between group activation differences (Colour scale red-yellow indicates T score, range 2.5-4).

**Figure 4:**
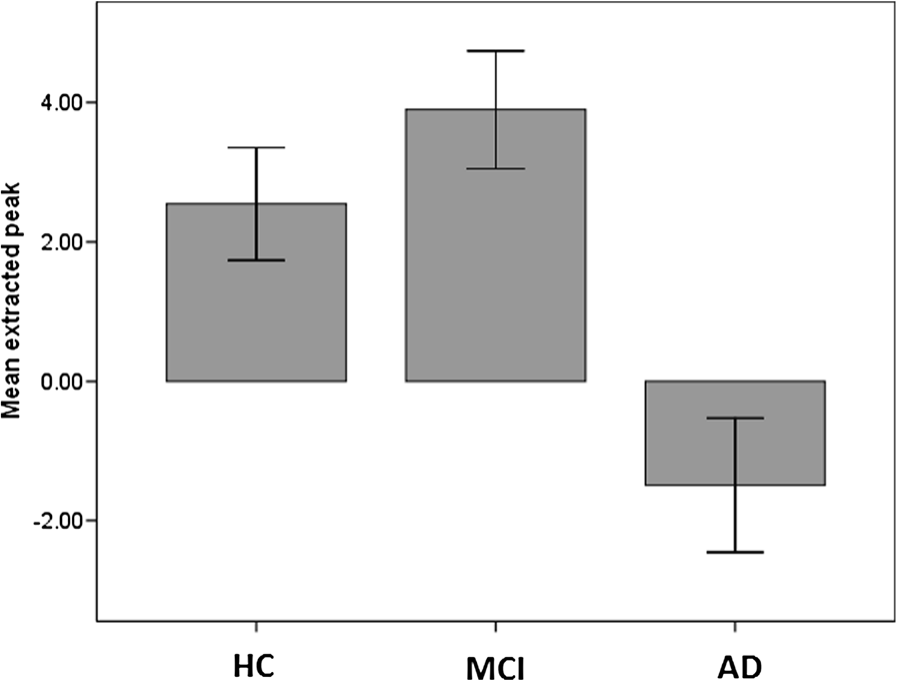
Between group activation differences for PHG/hippocampal cluster (error bars ± 1 SE).

Between groups results for all four contrasts examined are presented in Table 2. For the contrast of all scenes versus baseline, the AD group demonstrated decreased activation of the PHG/Hippocampal region versus both the controls (p = 0.06) and MCI group (p = 0.03) with the MTL small volume correction applied at threshold p < 0.001 uncorrected (see Figure 3 below). This pattern was also seen for the contrast of neutral scenes versus baseline, significant both against HC and MCI individuals. Again, a similar pattern of findings existed for the contrast of emotional scenes versus baseline but was below the chosen statistical threshold. There were no significant differences between the groups for the contrast of emotional scenes versus neutral. A graphical representation of the extracted data from the cluster of differences for the contrast of all scenes versus baseline is shown in Figure 4.

**Table 2:** Random effects analysis between groups

**Group**	**P value**	**K**_**E**_	**Z**	**Co-ords**	**Region**
**All scenes versus baseline**					
**HO>AD**	0.06	38	3.67	−28 -26 -16	Hippocampus/parahippocampal gyrus*
**MCI>AD**	0.03	73	4.25	−28 -26 -16	Hippocampus/parahippocampal gyrus*
**Neutral versus baseline**					
**HO>AD**	0.02	119	4.65	−30 -26 -16	Hippocampus/parahippocampal gyrus*
**MCI>AD**	0.02	106	4.85	−30 -26 -16	Hippocampus/parahippocampal gyrus*
**Emotional versus baseline : n/s** (but clusters in similar locations to above at lower threshold)					
**Emotional versus neutral : n/s**					

IQ and gender entered as covariates into the analysis. Analysis thresholded at p = 0.001 uncorrected.* = small volume correction for MTL applied. K_E_ = cluster size in voxels.

### Correlation with behavioural measures

VOI values from the three groups were extracted from the contrasts where MCI > AD in the MTL for each emotion versus baseline and neutral versus baseline separately. These contrasts were chosen because HC and MCI did not show significant differences in fMRI activation during the between-group analyses but both showed greater activation than the AD patients. These values along with corrected recognition data from the post-scan test obtained from the three groups were entered in a bivariate Pearson correlation analysis. VOI values in the MTL during the encoding of Emotional pictures significantly correlated with performance in the post-scan test for Emotional (r = 0.42, p = 0.02) and Neutral pictures (r = 0.37, p = 0.04). When only MCI and AD patients were entered in the analysis, the correlation patterns described above persisted (Emotional pictures: r = 0.55, p = 0.01; Neutral pictures: r = 0.46, p = 0.04).

## Discussion

The present study investigated whether functional and behavioural measures of incidental memory performance with emotional stimuli could help identify patterns of deterioration in MTL structures in MCI and AD patients. A profound decline in both functional and behavioural measures of episodic memory was found in the AD group relatively to MCI and HC.

Within-scanner behavioural measures showed response rates over 90% for each group. No significant differences across groups were observed for these measures. Similar results were found by Whalley et al. [25] with a much younger group of participants. This suggests that despite the incidental nature of the task, the arousal within the scanner remained high throughout the assessment for the three groups of participants regardless of the content of the stimuli. The post-scan test however yielded different results. Memory performance was found to be worse in the AD group than in MCI and HC groups. Post-hoc tests confirmed that HC was the only group that showed a significant effect of the emotional content of the stimuli. Previous studies have suggested that emotional memory deteriorates in patients with MCI [19,21] and AD [12-17]. However, incidental memory for emotional stimuli has been less well investigated in AD, particularly with the use of natural scenes. Previous studies reporting relatively preserved implicit functions in AD used eye movements recording [32] or tasks which did not have long intervals between encoding and test [31]. Our post-scan test was applied immediately after the scan. However, a few minutes lapsed in between these two phases. This may have led to decay of memory traces and attenuation of the facilitation elicited by the emotional content of the scenes in AD patients and to a lesser extent in MCI patients. However, HC could retain both more memory traces formed during the incidental task and the effects of the emotional pictures. The Socio-Emotional Selectivity Theory [50,51] posits that older adults’ increasing awareness of time limitations motivates them to prioritize the seeking of emotional meaning over other goals. This can result in them prioritizing more positive and emotionally significant experiences a process that seems to be affected by AD.

Increased fMRI activity was found in HC and MCI groups in both MTL and medial frontal regions for the specific contrast of all scenes vs. fixation baseline but not in the AD group. Increased activation of the hippocampus has been previously reported in MCI patients relative to cognitively intact individuals during associative memory tasks [52] and also in asymptomatic individuals who carry certain genotypes which lead to AD (e.g., E280A-PS1 [53] and APOE-e4 carriers [54]). Although fMRI differences between MCI and HC in our studies did not reach significance, the former group showed greater activation than the latter and was significantly different from AD patients. This seems to be in line with the suggestion of a compensatory phase early in the course of prodromal AD evidenced by increased MTL activation which is followed by a subsequent decrease as the disease progresses [52,55,56]. These memory-related changes in the hippocampal system might reflect a compensatory effort to overcome preclinical neural dysfunction caused by early pathological changes which do not reach threshold for behavioural expression. They suggest that hippocampal fMRI patterns during memory encoding may provide a preclinical biomarker for AD (see Quiroz et al. [53] for a similar view). Although our findings only hint to such mechanisms, future studies could investigate such a proposal using the present paradigm in a larger group of patients. Within and between group comparisons revealed that these activations were more significant for the contrasts of all scenes vs. baseline and emotional vs. baseline. A previous study which also used the emotional memory paradigm reported here suggested the usefulness of this methodology to investigate emotional-related decline in MTL structures in patients with schizophrenia and bipolar disorders [25]. However, they also acknowledged some limitations to this task. The authors claimed that positive scenes may be less uniformly arousing than negative emotional scenes. The block design used in the present study does not allow us to investigate whether the valence of some images selected from the IAPS was too subtle as to elicit differences in emotional processing at a functional level. The use of a block design may have resulted in the averaging of scenes of varying emotional intensity for a given subject within blocks. Furthermore, each block may include both remembered and forgotten pictures (see Whalley et al. [25] for a more thorough discussion on these issues) We thought it would be useful to consider these limitations as they could help us to better discuss our results within the current framework.

Reduced brain activation in MTL and frontal regions has been previously reported using explicit memory tasks in AD [27,57-60] and MCI [61,62]. However, the effects of emotional scenes on incidental learning in MCI and AD have not been thoroughly investigated using fMRI. As for the behavioural measures, the AD group showed the most impaired pattern of functional activation and together with the MCI group, they both showed no behavioural benefit derived from the emotional content of the stimuli. Potential accounts for these results were discussed above. An additional account for this functional impairment could be a poor encoding of emotional information. It might be possible that AD and MCI patients did not perceive the emotional content of the pictures as HC did. In fact, we screened this hypothesis in a subgroup of participants (5 HC, 7 MCI and 3 AD patients^b^) which were asked to rate the images after the post-scan test as emotional or non-emotional. AD rated as emotional only 37% of the scenes presented within this category whereas they rated as neutral 84% of the neutral scenes. MCI patients rated as emotional 60% of the scenes and neutral 66.9%. HC rated as emotional 75.5% and neutral 77.8%. However, the perceptual impairment observed in this small group of patients did not hold during the functional assessment of the whole group. As we suggested above, the subtle difference across the emotional categories for some of the images chosen for the present study may account for the lack of a differential impact on functional activation. In fact, correlation analyses with behavioural and neuroimaging data showed that the relation between MTL activation and successful performance in the post-scan test across the two set of images was significant. An alternative explanation for the current findings could be that MTL regions are relevant to the overall episodic memory component of the current task but less so to its emotional component. MTL regions may be part of the network involved in the episodic memory processing of emotional stimuli but structures outside the MTL region may be more specifically involved in the emotional processing. In fact, the actual contribution of MTL regions (e.g., amygdale and hippocampus) to memory for emotional materials is not well understood. Different aspects of emotional stimuli (i.e., valance and arousal) seem to be processed by different regions of this network [63-65]. For example, the effect that the amygdale exerts on the hippocampus during emotional processing appears to be modulated by the arousal more than by the valence of the stimuli. Stimuli with low arousal do not activate the amygdale-hippocampus network [64] even if their valence can be accurately recognised [63]. Our emotional stimuli have high valence (M = 7.21, SD = 0.51) but low arousal (M = 5.7, SD = 0.62). This may help explain the dissociation between behavioural and functional data in HC whereby effects of the emotional content was observed for the former but not for the latter data. AD appears to profoundly impact on the entire network which explains the lack of functional and behavioural changes across the two emotional dimensions as well as the dramatic drop in both measures relative to HC. MCI patients may recruit areas adjacent to the functionally impaired regions as a compensatory mechanism [66]. These mechanisms would support the episodic component of the present task but not the emotional component. As for the AD group, the MCI patients failed to show emotional effects at both behavioural and functional levels even though their episodic memory was not poorer than that of HC.

The evidence provided here warrants further investigation on this subject. The fact that both AD and MCI patients showed behavioural impairment in the emotional memory task but only the former group showed neuroimaging changes suggests that contrary to episodic memory, deterioration of emotional memory in these two conditions may have different neural correlates and may follow different trajectories (i.e., MCI may not always be preclinical AD, see Albert et al. [41]). These preliminary results in a small sample of MCI patients suggest that investigating the accuracy with which combined behavioural and neuroimaging data with the emotional memory paradigm could predict MCI to AD conversion would be worthwhile. Future studies may incorporate stimuli with negative valence or positive stimuli with higher arousal values. We predict that such manipulations would not undermine our primary findings, but they may shed new light on the actual involvement of the MTL network during emotional memory processing in AD and MCI. Moreover, in the light of the limited number of studies available which have investigated emotional memory processing in MCI and AD using fMRI, we believe that the results presented here will stimulate future research on this subject. Neuroimaging biomarkers are becoming more reliable and widely incorporated in decision making algorithms for the clinical diagnosis of dementia [67]. When behavioural and functional (fMRI) measures of intentional memory are used, MCI and AD tend to show a great overlap [59] even though not all MCI patients convert to AD. This largely reflects the age-related decline of MTL structures. The ability to process arousing stimuli however seems to be preserved in old age [68]. Here we have shown that incidental emotional memory may help reduce the MCI/AD overlap. As we discussed above, this distinction might be disclosing age-unrelated cognitive decline which more reliably inform when MCI may be preclinical AD. Combining fMRI and the emotional memory paradigm reported here may offer a valuable tool for the preclinical assessment of risk for AD (see Reiman et al. [69] for recent evidence in familial AD).

Some limitations of the present study were discussed above (i.e., related to the stimuli used and the fMRI design). Another limitation is the sample size. Ten participants per group was enough to identify marginal effects in the two-way ANOVA with behavioural data (p = 0.074, Power = 52%). It is worth noting that conducting fMRI studies with patients with AD is very challenging. This may be a factor explaining the limited number of studies available on the subject. Larger sample sizes will allow a better characterization of the effects of MCI and AD on emotional-related memory processing and MTL structures. Additionally, the use of an incidental memory paradigm in an fMRI environment with vulnerable older adults such as those with dementia may be other factors to be considered when designing fMRI studies. The effects of the fMRI environment on cognitive performance are now better understood [70]. Future studies might use scanner simulators to investigate these effects and to prepare the research participants prior to fMRI assessment.

## Conclusions

Important clusters of activation were observed with the emotional memory paradigm in HC and MCI in MTL and medial frontal regions. These clusters were not observed in AD. AD and MCI patients showed behavioural impairment in the emotional memory task but only the former group showed neuroimaging changes. This suggests that contrary to episodic memory, deterioration of emotional memory in these two conditions may have different anatomical, functional and clinical implications. Whether a combined analysis of behavioural and neuroimaging data with the emotional memory paradigm could yield better predictive power to assess conversion to AD among MCI patients is subject which will require investigation.

### Endnotes

^a^This task was the result of several pilot sessions. AD patients found it difficult or confusing to perform within the scanner versions of this memory task which required old/new responses or which assessed several memory processes (i.e., encoding, maintenance and retrieval).

^b^After we had assessed a few participants, we noticed that patients, particularly of the MCI group, did not perceive all the emotional stimuli as such. Hence, we decided to test perception of our stimuli in the last participants recruited into the study.

## Competing interests

HCW, AMM, SML and JH were in receipt of grant funding from the Translational Medicine Research Collaboration, described above. HIW and GH were employees of Wyeth, now Pfizer. The other authors have no biomedical financial interests or potential conflicts of interest.

## Authors’ contributions

MAP: has made substantial contributions to acquisition, analysis and interpretation of data; has been involved in drafting the manuscript and revising it critically for important intellectual content; VP, DW, AB, JL: have made substantial contributions to acquisition and interpretation of data and have been involved in revising the manuscript critically; HIW, JH: have made substantial contributions to conception and design and have been involved in revising the manuscript critically for important intellectual content; HCW, SML: have made substantial contributions to conception and design, analysis and interpretation of data, have been involved in drafting the manuscript and revising it critically for important intellectual content; GH: has made substantial contributions to the interpretation of data, has been involved in revising the manuscript critically for important intellectual content. All authors read and approved the final manuscript.

## Pre-publication history

The pre-publication history for this paper can be accessed here:

http://www.biomedcentral.com/1471-244X/13/76/prepub

## Supplementary Material

Additional file 1 Figure S1 All scenes versus baseline (Threshold p < 0.001). **Figure S2.** Contrast for the emotional versus neutral scenes (Threshold p < 0.001). Click here for file
