# Supplementary material for: Medial temporal lobe function during emotional memory in early Alzheimer’s disease, mild cognitive impairment and healthy ageing: an fMRI study
Source: BMC Psychiatry. 2013 Mar 6;13:76. doi: 10.1186/1471-244X-13-76 (PMC3599533; doi:10.1186/1471-244X-13-76)

**Supplementary Material**

**Figure 1.** All scenes versus baseline (Threshold p < 0.001).


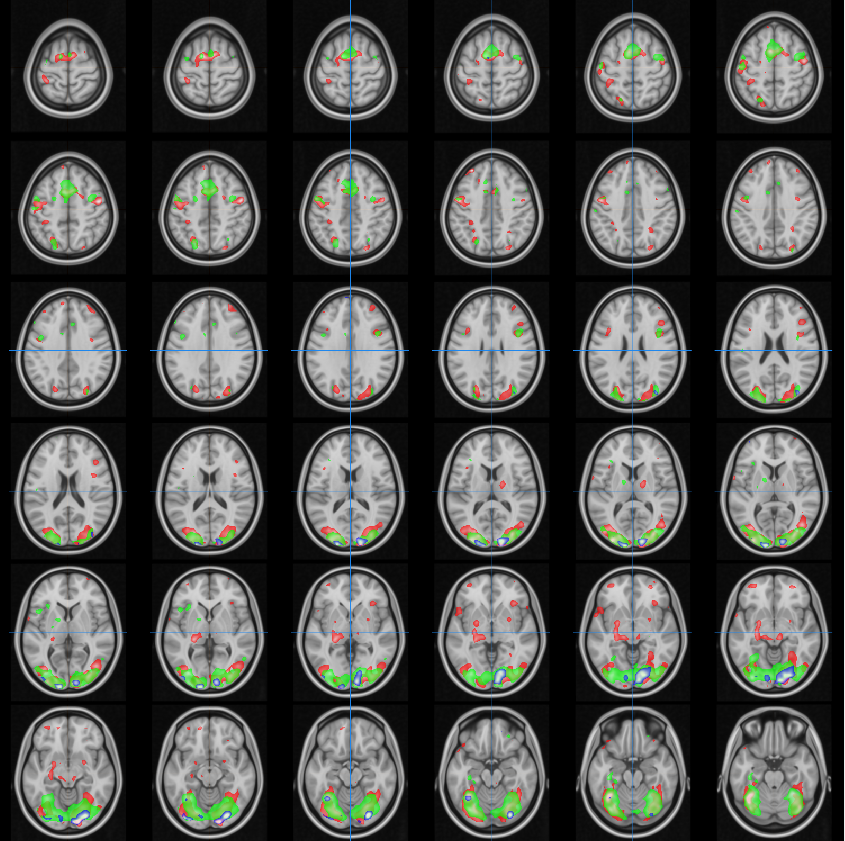


**Figure 2.** Contrast for the emotional versus neutral scenes (Threshold p < 0.001).


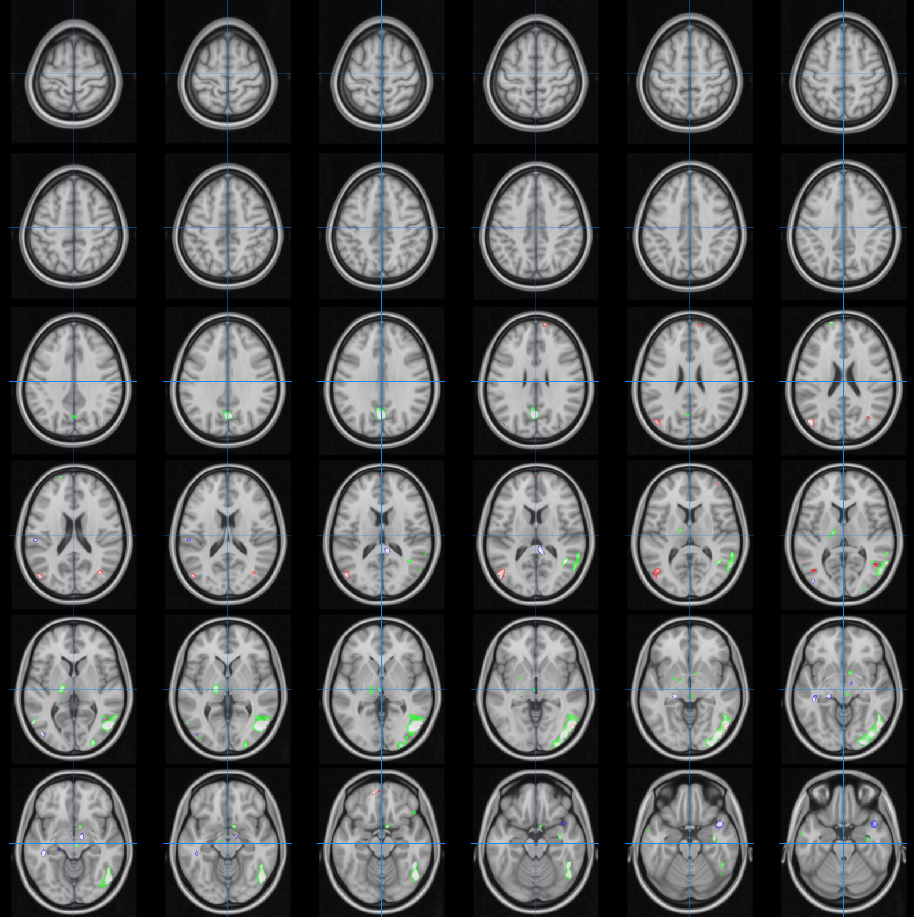

Supplement: Additional file 1 Figure S1 — All scenes versus baseline (Threshold p < 0.001). Figure S2. Contrast for the emotional versus neutral scenes (Threshold p < 0.001). [file 1471-244X-13-76-S1.doc]
